# Knowledge of Cervical Cancer Prevention Among Women in Amazonian Peru

**DOI:** 10.1089/whr.2020.0051

**Published:** 2020-08-17

**Authors:** Lauren Gochenaur, Sara Peterson, Luis Vasquez, David Adler

**Affiliations:** ^1^ School of Medicine and Dentistry, University of Rochester, Rochester, New York, USA.; ^2^ Yantalo Peru Foundation, Yantalo, Peru.; ^3^ Emergency Medicine & Public Health Sciences, University of Rochester Medical Center, Rochester, New York, USA.

**Keywords:** HPV, Peru, cervical cancer, Papanicolaou, knowledge

## Abstract

***Background and Purpose:*** Survey-based research was conducted in Yantalo, Peru, a rural Amazonian community, to assess the knowledge base among women surrounding cervical cancer, human papilloma virus (HPV), and preventative health practices as well as to gain a better understanding of barriers to accessing care.

***Methods:*** A total of 217 women were interviewed out of the 1612 female inhabitants of Yantalo utilizing a structured interview-style questionnaire with both closed and open-ended questions.

***Results:*** Our average respondent was 41.6 years old with the equivalent of some high school education. Approximately 75% of respondents reported that they had heard of HPV and/or cervical cancer, with 44.4% reporting they had received a test to check for cervical cancer within their lifetime. When given a 10-question knowledge assessment regarding safe sex practices and cervical cancer, women obtained an average score of 57.3%. When asked about receiving the HPV vaccine, 29% reported “Yes”, 59.4% reported “No”, and 11.6% reported “I don't know.” Although 62.6% of women indicated that they have “easy access to cervical cancer screening,” 37.4% of women reported experiencing at least one barrier to accessing care. The highest reported barriers include fear of the test causing them pain and/or lack of knowledge of the necessity cervical cancer testing. Cervical cancer rates in Peru are approximately three times that in developed countries.

***Conclusions:*** Gathering data surrounding knowledge and the barriers among the female population in rural communities is essential to developing targeted initiatives that address pertinent obstacles within these and other vulnerable communities.

## Introduction

Globally, cervical cancer is the second most common cancer in females.^1^ Cervical cancer has been identified as the largest cause of potential years of life lost in the developing world.^2^ The overall age-standardized rate (ASR), or incidence per 100,000 people, for cervical cancer in South America is estimated to be 20.4. Peru holds the fourth highest ASR in South America at 22.2, behind El Salvador, Bolivia, and Guyana, and has a mortality rate of 9.4 per 100,000 women.^3^ In comparison, the Center for Disease Control (CDC) reports the ASR for the United States to be 7.7 per 100,000 women.^4^ Lima, Peru, which is located on the coast, reported an ASR of 19.2, whereas less-populated cities such as Trujillo in the north and Arequipa in the south both reported an ASR of 43.2.^5^ These differences are suspected to be due to disparities in education and access to health care services. Other than the three cities stated earlier, no other cancer registries exist in Peru.^5^

Peru's Ministry of Health recommends screening for women aged 30–49 years with Papanicolaou (Pap) tests, and/or visual inspection with acetic acid (VIA).^2^ Pap test screening coverage is estimated to be 42% in rural areas compared with 57% in urban areas.^6^ In addition, a multivariant analysis revealed that women who received Pap testing were more likely to be married with at least two children, have received higher education, and hold residence in a coastal area.^7^ In Peru, barriers to cervical cancer screening include lack of availability of trained staff, lack of follow-up, as well as economic, geographical, and educational barriers.^6^ Along with testing for cervical cancer, vaccinating against oncogenic human papilloma virus (HPV) genotypes 16 and 18 has proven effective in decreasing one's risk for cervical cancer significantly.^8^ HPV vaccination is currently licensed in Peru for women aged 9–26 years.^9^ HPV vaccination started in Peru in 2008, primarily through school-based HPV vaccination campaigns.^10^ Considering the high incidence and mortality of cervical cancer in rural Peru, gathering data surrounding knowledge and barriers to preventative care is instrumental in developing focused initiatives to reduce cancer rates in the future.

**FIG. 1.**
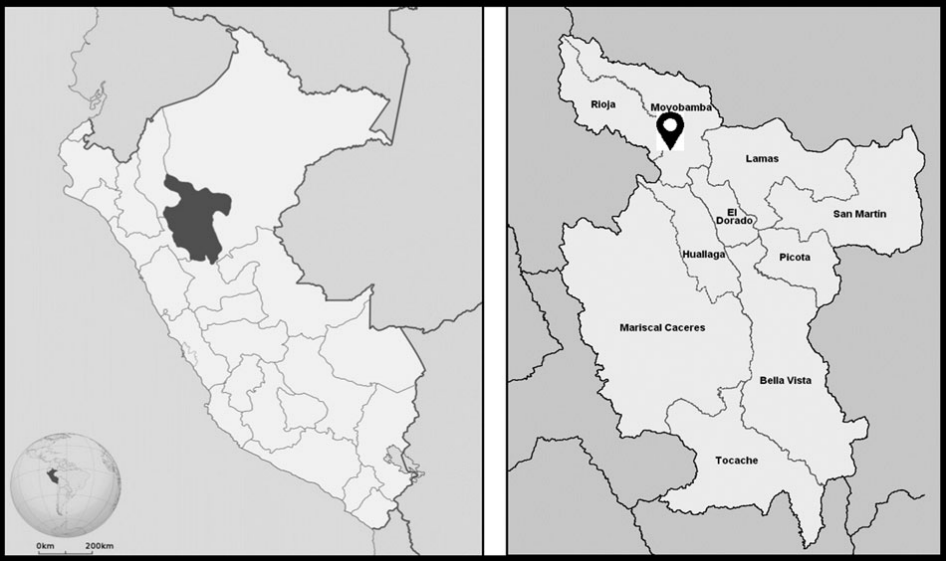
Geography of Peru: map of Peru with the San Martin region highlighted (left); provinces of San Martin region with arrowhead indicating Yantalo, Peru (right).

Yantalo is a town located within the Peruvian Amazon, a historically isolated and impoverished region within Peru (Fig. 1). Yantalo has 3535 inhabitants, where 1612 (45.6%) are female and Spanish is the primary language.^11^ Approximately 50% of households live in poverty in the Peruvian Amazon.^12^ In addition, 20% of the population in the Peruvian Amazon reports they have never seen a doctor, citing the primary reason as financial constraints.^12^ Women's health care is free and accessible at a small medical post, the Center of Health, in the central town square of Yantalo. This post is usually short-supplied and understaffed when compared with the large patient volume; however, Pap tests and acetic acid testing are offered there. In Yantalo, HPV vaccination is only available through periodic government-sponsored campaigns.^11^ Currently, there are no statistics on HPV vaccination rates in Yantalo.^11^ The only HPV vaccination rates in the Amazon region of Peru found in literature review are within Iquitos, who report 98%, 88%, and 65% of girls receiving the first, second, and third doses, respectively.^5^

## Objectives

Researchers worked in conjunction with the Yantalo clinic and local municipality to collect data regarding the awareness and knowledge of HPV and cervical cancer with the goal of guiding future community health education initiatives. In addition, researchers also assessed barriers to accessing cervical cancer screening care in Yantalo.

## Materials and Methods

We designed a survey to assess content knowledge and barriers to accessing preventative care in Yantalo. The questionnaire consisted of four close-ended question sections: demographic information, gynecological health history, barriers to care screening, and a knowledge assessment, as well as one open-ended section allowing women to voice questions and offer input on how to improve knowledge in their community regarding their personal experiences and attitudes (Supplementary Appendix SA1). Similar questionnaire-based HPV knowledge assessments have been previously utilized in resource-limited settings in Peru with success.^13^ Assessment of attitudes regarding HPV vaccination in Peru has previously been done using a guided interview technique with pattern-based theme recognition.^10^ The questionnaire developed for the Yantalo community contained the option for open-ended input that utilized similar transcription of text and systematic recognition of similar themes.^10^ The questionnaire developed for the Yantalo community contained a true/false knowledge assessment portion that was based upon a previously validated measure, which was utilized in a resource-limited setting.^14^ The survey was translated by a Peruvian native into Spanish, then reviewed and approved for cultural context by medical staff at the Yantalo clinic. Notably, common English acronyms/abbreviations were not used in the Spanish version of the survey, that is, Pap tests were referred to as “examen Papanicolaou,” and HPV was termed “virus del papiloma humano.”

The method for disseminating surveys was a collaborative effort between Dr. Vasquez, Yantalo municipality leadership, and the study team members. The researchers were given a detailed community plat map that outlined the land divisions of each household, allowing researchers to systematically interview each household. Yantalo has a population of 1612 women (46.5% of the population), 986 of which were >21 years and eligible to participate in the survey.^11^ A total of 217 women (21% of our target population) were successfully surveyed. Study team members went door to door in the community each day disseminating surveys in the native language, Spanish. The study team completed two rounds of surveys dissemination, one in the morning and one in the early evening. Subjects were eligible if they were women at least 21 years of age, and from the Yantalo community. Detailed inclusion and exclusion criteria are summarized in Table 1. The survey was administered using a structured interview format to ensure women of all literacy levels were able to participate. Administering one survey took ∼10–15 minutes. After completing the survey, respondents were asked were given a fact sheet designed by the CDC (Supplementary Appendix SA2) with risk factors, symptoms, and available testing methods for HPV and cervical cancer and provided the opportunity to ask any additional questions.

**Table 1.** Survey Inclusion and Exclusion Criteria

Inclusion criteria	Exclusion criteria
Female gender identity	Unable to provide informed consent
Ages >21 years	Does not speak Spanish or English
Citizen of Yantalo, Peru	Has previously completed the survey

Each question within the survey's knowledge section had an option of “true,” “false,” or “I don't know.” A response of “I don't know” was coded as incorrect, as it indicated a lack of knowledge. Surveys did not ask participants for personal identifiers and participants had the option to discontinue the survey at any time. Survey responses were recorded in Spanish and translated into English by the authors. When unfamiliar regional dialect terms were used, translation was verified by bilingual staff at the Yantalo Clinic. Paper surveys were entered into a password-protected electronic database with access limited to research staff. Data were analyzed in Prism GraphPad software using the analysis of variance (ANOVA) tool, Bonferroni *post hoc* analysis, and confidence interval features.

### Ethical statement

We obtained verbal consent from all women who agreed to participate in the study. Researchers gave potential participants a synopsis of the study, estimated time commitment, option to opt out at any time and reassured that their participation status in this survey would not impact their ability to access health care in any way. Researchers requested and received study approval as well as exemption from documentation of consent from the Research Subjects Review Board at the University of Rochester Medical Center. This project was approved by the Yantalo Clinic, Yantalo Health Center, and municipal leadership.

## Results

Data from 217 respondents were collected, and 10 surveys were excluded from data analysis due to failure to meet inclusion criteria, leaving 207 surveys included in data analysis. Fifty-six percent of respondents identified having completed primary school (equivalent to elementary school in United States) but not secondary school (equivalent to high school in United States). 75.8% of respondents (*n* = 157) reported having heard of HPV, whereas only 23.7% (*n* = 49) reported knowing anyone diagnosed with HPV. Similarly, 77.8% (*n* = 161) of respondents reported having heard of cervical cancer, whereas 33.3% (*n* = 69) reported knowing someone diagnosed with cervical cancer. When women were asked if they had ever received a test to check for cervical cancer, 44.4% (*n* = 92) reported that they had.

When assessing barriers to care (Table 2), 62.6% (*n* = 129) of women responded yes “I am able to access cervical cancer screening easily” whereas 37.4% (*n* = 77) of women reported facing at least one barrier to accessing screening care. The barriers most reported were fear of pain during the examination (14.6%, *n* = 30), lack of awareness of the need for screening (12.6%, *n* = 26), and expense of the examination (9.2%, *n* = 19). Other barriers noted include transportation (8.7%, *n* = 18), distrust of medical providers (8.7%, *n* = 18), lack of access to nearby providers (6.8%, *n* = 14), and religious objections (5.3%, *n* = 11).

**Table 2.** Reported Barriers to Accessing Preventative Cervical Cancer Care

Barrier to care	Women reporting this as a barrier
Women indicated they have easy access to cervical cancer screening	129 (62.6%)
“I am afraid the test will cause me pain”	30 (14.6%)
“I didn't know cervical cancer testing is something I should have done”	26 (12.6%)
“The test is too expensive or I don't want to spend money on it”	19 (9.2%)
“I am distrusting or feel uncomfortable around medical providers”	18 (8.7%)
“It is hard for me to find transportation”	18 (8.7%)
“There aren't any providers nearby”	14 (6.8%)
“I have a religious or moral objection to obtaining a screening test”	11 (5.3%)
Other reasons (open-ended)	12 (5.8%)

Respondents were asked to identify whether they can obtain easy access to Pap testing or not. If not, possible barriers were presented in a “check all that apply” format.

Pap, Papanicolaou.

**FIG. 2.**
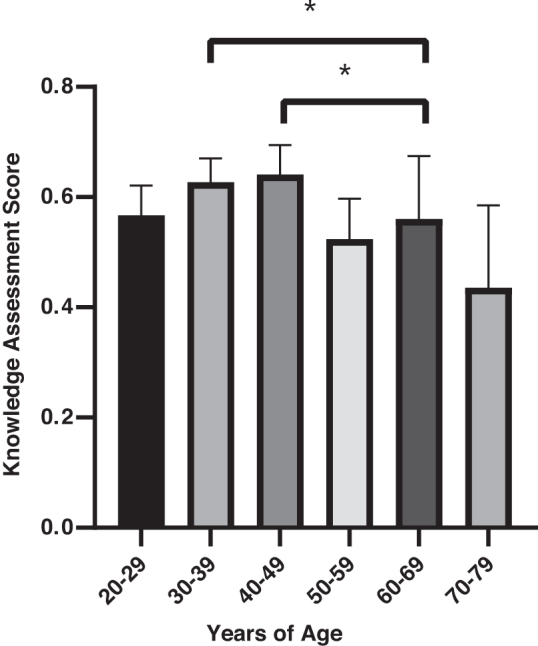
Knowledge assessment score stratified by age group: women between 30–39 and 40–49 years received the highest scores on the knowledge assessment. One-way ANOVA was significant *F*(6, 194) = 4.11, *p* ≤ 0.001. A *post hoc* ANOVA test revealed that women 30–39 (*M* = 62.7, SD = 15.6), *F*(13, 51) = 2.75, *p* = 0.001 and 40–49 (*M* = 64.1, SD = 15.2), *F*(13, 33) = 2.90, *p* = 0.013 years of age scored significantly higher compared with women 70–79 years of age (*M* = 43.7, SD = 25.9). Error bars represent 95% CI. Asterisk indication: *p < 0.05. 80+ age group was excluded due to low validity (*n* = 3). ANOVA, analysis of variance; CI, confidence interval.

**FIG. 3.**
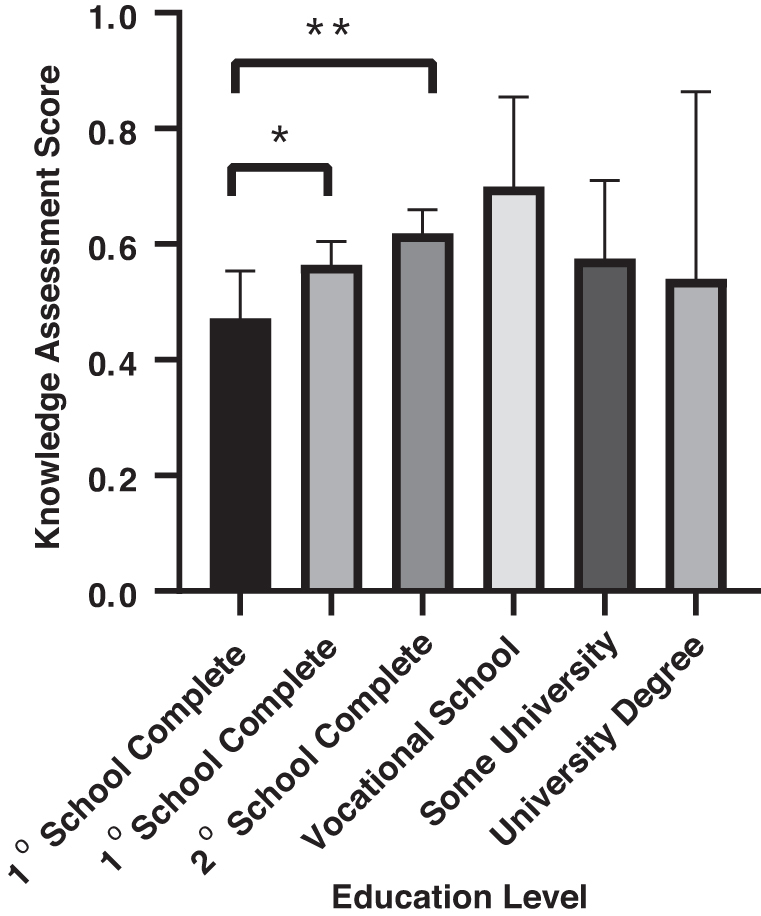
Knowledge assessment score stratified by education level: women with vocational school degrees performed the highest, with an average 70%. One-way ANOVA was significant *F*(6, 164) = 2.51, *p* ≤ 0.05. A *post hoc* ANOVA test revealed women who did not complete primary school scored significantly lower (*M* = 47, SD = 22) than women who completed secondary schooling (*M* = 61, SD = 16), *F*(31, 80) = 1.545, *p* = 0.025 and those who completed vocational school (*M* = 70, SD = 18), *F*(7, 31) = 1.49, *p* = 0.012. Error bars represent 95% CI. Asterisk indication: *p < 0.05, **p < 0.01.

When given a 10-question knowledge assessment, women had an average score of 57.3% (Table 3). When stratified by age, women between 30–39 and 40–49 years received the highest scores on the knowledge assessment, whereas women aged 70–79 years and 80+ performed the worst. Performance on the knowledge assessment was significantly different when stratified among age groups using a one-way ANOVA (*F*(6, 194) = 4.11, *p* ≤ 0.001) (Fig. 2) and education level *F*(6, 164) = 2.51, *p* ≤ 0.05 (Fig. 3). *Post hoc* test revealed that women 30–39 (*M* = 62.7, SD = 15.6), *F*(13, 51) = 2.75, *p* = 0.001 and 40–49 (*M* = 64.1, SD = 15.2), *F*(13, 33) = 2.90, *p* = 0.013 years of age scored significantly higher compared with women 70–79 years of age (*M* = 43.7, SD = 25.9). Similarly, women who did not complete primary school scored significantly lower (*M* = 47, SD = 22) than women who completed secondary schooling (*M* = 61, SD = 16), *F*(31, 80) = 1.545, *p* = 0.025 and those who completed vocational school (*M* = 70, SD = 18), *F*(7, 31) = 1.49, *p* = 0.012.

**Table 3.** Cervical Cancer Knowledge Assessment in Rural Peruvian Women

Question	True	False	I don't know
HPV causes cervical cancer	**137 (67.5%**)	13 (6.4%)	53 (26.1%)
HPV can be passed through sexual contact	**151 (74.4%)**	18 (8.8%)	34 (16.7%)
Using condoms reduces HPV transmission risk	**125 (61.2%)**	35 (17.2%)	44 (21.6%)
Having multiple sexual partners increases HPV contraction risk	**161 (78.9%)**	16 (7.8%)	26 (12.7%)
A vaccine can protect you from HPV	**118 (57.8%)**	14 (6.9%	72 (35.3%)
HPV vaccines can effectively prevent cervical cancer	**110 (53.9%)**	13 (6.4%)	81 (39.7%)
Pap smears and acetic acid testing check for cervical cancer	**149 (73.0%)**	8 (3.9%)	47 (23.1%)
Pap smears and acetic acid testing check for STIs	150 (73.5%)	**19 (9.3%)**	34 (16.7%)
Cervical cancer can be prevented by Pap smears	159 (77.9%)	**11 (5.4%)**	33 (16.2%)
Cervical cancer can be cured if detected early	**189 (92.6%)**	5 (2.5%)	10 (4.9%)
Spirits and supernatural can affect someone's health	100 (50%)	63 (31.0%)	39 (19.2%)

Correct responses are indicated in bold.

Knowledge was assessed using a multiple-choice question format with three possible answers, “Yes”, “No”, or “I don't know.” Women were asked an additional question pertaining to their beliefs of the supernatural as they relate to one's health.

HPV, human papilloma virus; STI, sexually transmitted infection.

When women were questioned about their individual HPV vaccination history, 29.0% (*n* = 60) reported “Yes” they had received the vaccine, 59.4% (*n* = 123) reported “No,” and 11.6% (*n* = 24) reported “I don't know.” Regarding free cervical cancer screening, 79.1% (*n* = 163) of the women reported “Yes” they would accept a free cervical cancer screening test if offered, whereas 12.6% (*n* = 26) reported “No,” and 7.3% (*n* = 15) reported “Maybe.”

In the optional open-ended section, women were asked how to increase education surrounding cervical cancer and HPV in Yantalo. 18% (*n* = 37) of women indicated that “charlas” (community talks) are an effective method of spreading information in their community. When asked if they had additional questions regarding the subject matter, common responses included, “What are the symptoms of cervical cancer?,” “How do you prevent cervical cancer?,” and “How do you treat HPV and cervical cancer?”

## Discussion

Researchers surveyed 21% (*n* = 207) of our target population of eligible adult women (*n* = 986) with the primary objectives of assessing the community's prevention-based HPV and cervical cancer knowledge and collecting data on cervical cancer screening rates and access in Yantalo, Peru. The average respondent was female, median age of 41.6 years, with the education equivalent of some high school.

### Familiarity with cervical cancer and HPV among Yantalo's women

The incidence rate of cervical cancer in Peru is 25.2%.^2^ 77.7% (*n* = 161) of women surveyed had heard of cervical cancer, whereas only 33.3% (*n* = 69) of respondents knew someone with a current or past cervical cancer diagnosis. When asked similar questions about HPV, 75.8% (*n* = 157) report having heard of HPV, and 23.7% (*n* = 49) of women report having knowledge of someone with a current/prior diagnosis of HPV. Considering the causal nature of the virus with cervical cancer, and the high prevalence of HPV,^2^ this suggests a need for education regarding these topics.

### Pap knowledge inconsistencies

Interestingly, there were inconsistencies between women responding “yes” to “Have you ever gotten a test to check for cervical cancer?” (44.4%, *n* = 92) and the number of women reporting when their most recent Pap test was 68%, (*n* = 141). This inconsistency suggests that women are unaware that Pap tests are in fact tests that check for cervical cancer. This is further supported in the knowledge assessment, where 23.1% (*n* = 48) of women responded “I don't know” when given the statement “Pap tests detect cervical cancer.” In a separate question, 77.9% (*n* = 161) of women incorrectly responded that cervical cancer can be prevented by receiving Pap examinations. In addition, 73.5% (*n* = 150) of women incorrectly reported that Pap tests are a form of testing for sexually transmitted infections (STIs). These findings suggest that women are often not aware that Pap tests are included in routine pelvic examinations and performed to screen for HPV and cervical cancer. Also, Pap tests and STI testing are often performed during the same visit/examination, which could explain the misunderstanding of what these examinations are testing for. This suggests that information about Pap tests and STI screening should be made available to the women of Yantalo to clarify these misunderstandings.

### The knowledge assessment

The average score on the knowledge assessment was 57.3%. The finding that younger women (aged 20–49 years) obtained higher scores on the knowledge assessment than older women (aged >70 years) may be due to increased number of women attending schools, improved reproductive/sexual health education in schools, and Internet accessibility. Presence of reproductive health programs in school may also explain why women in the lowest scoring group were those who did not complete primary school. Only 5 women with college bachelor's, master's, or doctorate (2.4%) and 13 women with some university education (6.3%) were surveyed. It is recognized that women who hold higher levels of education may not reside in Yantalo due to the lack of career opportunities. Specific data regarding population education level are not available from the Yantalo Municipality. 67.7% (*n* = 137) of women surveyed responded correctly when asked if HPV causes cervical cancer, whereas a 2012 study conducted in Cusco, Peru, reported that 38% of women reported correctly recognizing that HPV causes cervical cancer in a similar questionnaire.^15^ This suggests an increased awareness and knowledge about cervical cancer and HPV among women within the past decade, which may be attributable collaborative campaigns between the Program for Appropriate Technology in Health, global nonprofit focused on preventative nonprofit health care initiatives and the Peruvian government.^9^

### HPV vaccination rates

59.4% (*n* = 123) of respondents reported that they personally had not received the HPV vaccine within their lifetime, with only 29.0% (*n* = 60) reporting “yes” to having personally received the vaccine. However, “Yes” respondents may be overestimated due to prevalent vaccine misunderstandings among women. When considering that HPV vaccination in Peru is available to women ages 9–26 years and began in 2008, the oldest women who theoretically should have personally received the vaccine would be 37 years old at the time of survey. There were 27 women (45% of Yes respondents) >37 years who potentially falsely reported that they had received an HPV vaccine, which may be attributed to confusion of vaccination history. For some women it has been >10 years since vaccination and recall issues may impact reliability. Furthermore, misconceptions about what vaccines cover were observed. For example, some women during survey administration would respond “yes” to receiving HPV vaccines, and then mention another illness such as rubeola or influenza, even when reiterating specifically for the HPV vaccine.

### Comparison of center of health versus collected data

Our survey data report 41% (*n* = 85) of women have received cervical cancer screening within the past year. Data from the Yantalo Center of Health report that 118 Pap tests and 104 VIAs were done within the past year, for a total of 22.5% of women receiving cervical cancer screening.^11^ This number is likely underestimated, as women from Yantalo often travel to Moyobamba, a neighboring city, for pay-per-service gynecological care. In the San Martin region, where Yantalo is located, estimated screening coverage of the general female population is 60.1%.^2^ In Peru, cervical cancer screening is recommended in women ages 30–49 years *via* Pap or VIA every 3 years.^2^ Data from the Yantalo Center of Health show that women aged 30–49 years receive VIA testing and women of 50–65 years more commonly receive Pap tests.^11^ Notably, women requiring further testing after an abnormal Pap test are referred to Tarapoto, a larger city 2 hours from Yantalo, for cryotherapy testing. Cryotherapy testing capability was brought to Taropoto by the Pan American Health Organization in 2000 and currently remains a referral center.^16^

### Barriers to care

Two commonly reported barriers to care were fear of pain during a Pap test (14.6%) and not knowing that Pap tests are recommended cancer screening for Peruvian women (12.6%). Both of these findings are consistent with prior studies of barrier assessment,^5,15^ and this indicates that education campaigns could be directly useful in decreasing these specific barriers. Furthermore, expense of the examination (9.2%), transportation (8.7%), and lack of access to nearby providers (6.8%) were reported as barriers to care. Although the examination is free at Yantalo's Center of Health, it is acknowledged that there is a shortage of women's health providers, the available providers face a volume of patients that is above facility capacity, and that wait times for visits are long. This shortage of women's health providers reported in the Peruvian Amazon is consistent with prior literature,^17–20^ as is the high cost.^12,20^ Moyobamba is a local city with a higher volume of women's health providers with increased availability, but many of these health facilities charge a fee per visit and transportation costs to Moyobamba must also be considered. In addition, 8.7% of women reported a distrust of medical providers as a barrier to their cervical cancer screening care. Anecdotally, women attributed this distrust due to doctors frequently “not able to do anything” for illnesses of loved ones, strong beliefs in “curanderos” (traditional healers) and traditional medicine, and previous negative experiences with doctors. It has been reported in a similarly rural town within the Peruvian Amazon that 17% of participants were more likely to be compliant with traditional medicine rather than modern medicine,^17^ and that 47% of the population reported use of traditional medicine.^19^ In addition, in the Peruvian Amazon, a prior study reported that women were less likely to seek health care due to reluctance to be examined by a male doctor.^17^

### Limitations

It is a notable limitation that our survey performed in South America was created based on a measure previously validated for use in Africa. To our knowledge, similar HPV knowledge assessments have not been performed in rural Peru. As detailed in methods, we met with clinic staff to verify our translation to Spanish and ensure appropriate cultural contexts. Despite this, we recognize that cultural differences exist, and this may impact the validity of our results. An additional limitation could be the inclusion of both terms “cuello uterino” and “cervical” in the survey. Researchers had hoped to increase recognition by including both terms, but often had to explain to women during survey administration that the terms were synonymous. It is admitted that this could have led to confusion and inaccurate answers. Other concerns include unavoidable subjective terminology such as “easy access to care” when contrasting reportable barriers to care. Further explanation to the intended meaning of easy access was available as needed. In addition, the surveys were conducted in Spanish, which is not the native language of the researchers. Regional slang terms were used by women, which required clarification during interviews and verification with bilingual local clinic staff. The survey was translated into Spanish, and results were translated into English, which poses a risk of cultural contexts and meanings being lost in translation. It is recognized that the distinction between prevention, detection, and screening may not be as specific in Spanish as in English, and this nuanced difference may have been a point of confusion and/or lost in the translation process.

### Future directions

79.1% (*n* = 163) of women reported that they would accept a free test for cervical screening, whereas only 69% (*n* = 142) of women received their recommended Pap examination within the past 3 years. Holding cancer screening campaigns may be an effective way to increase screening rates within the recommended time frame.

When considering barriers to care, public health initiatives that dissuade fear of receiving a Pap test, education about what Pap examinations detect, and increase awareness of cervical cancer prevention may be key in increasing rates of cervical cancer screening in Yantalo. Education materials that directly describe a Pap test could ensure women know what to expect during their visit. In addition, education that emphasizes the link between HPV and cervical cancer, that cervical cancer is detected *via* Pap test, the annual screening recommendations for women in Peru, and that earlier detection results in increased treatment outcomes could decrease unawareness surrounding screening recommendations. It has been previously reported in the Peruvian Amazon that health education strategies were successful in improving health outcomes, although this education was surrounding helminth infections and their effect notably was not long-lasting.^21^

Reducing the costs of screening tests and decreasing the need for transportation could be achieved by expanding services at the free Center of Health in Yantalo. The recruitment of additional women's health providers, expansion of the days that women health providers see patients, or training/encouragement of primary care providers at the Yantalo Center of Health all could expand access within the free clinic.^18^ These results could be presented to local governments to increase funding to the health center, which may ease the financial burden faced by the Center of Health.

The results of this research were shared with the Yantalo Clinic, the Yantalo Center of Health, and the Municipality Leadership of Yantalo. An informational PowerPoint presentation about HPV and Cervical Cancer, which targeted the known gaps of knowledge, barriers to care, and common misconceptions was created in Spanish and shared with the clinic staff. In addition, the authors provided the clinic with the CDC Information Sheets on HPV and Cervical Cancer for dissemination. Furthermore, women's self-reported HPV vaccination rates were shared with the Center of Health, providing the first recorded public health data on this subject.

## Conclusions

Women in Yantalo are aware of HPV and cervical cancer, yet knowledge lags behind awareness. Knowledge campaigns and community talks elaborating on Pap tests, HPV, and cervical cancer would be helpful ways to disseminate information and increase community knowledge in rural Peru. Sharing information about Pap tests may help women understand why the examinations are performed and dispel fears held about the examination, appropriately addressing the two most reported barriers.

## Supplementary Material

Supplemental data

## Supplementary Material

Supplementary Appendix SA1

Supplementary Appendix SA2

## Abbreviations Used

ASR: age-standardized rate
ANOVA: analysis of variance
CDC: Center for Disease Control
HPV: human papilloma virus
Pap: Papanicolaou
STI: sexually transmitted infection
VIA: visual inspection with acetic acid
