# Supplementary material for: Knowledge of Cervical Cancer Prevention Among Women in Amazonian Peru
Source: Womens Health Rep (New Rochelle). 2020 Aug 17;1(1):270–8. doi: 10.1089/whr.2020.0051 (PMC7784820; doi:10.1089/whr.2020.0051)
Supplement: Supplemental data [file Supp_Data.zip › Gochenaur&Peterson.AppendixI.docx]

**Demographics**

1. How old are you? ________ **years**
2. Please check off the highest level of schooling that you have completed.
   - Less than Primary School
   - Completed Primary School
   - Completed Secondary School
   - Vocational School
   - Some college
   - Bachelor’s, Master’s, Doctorate, or other graduate level degree

3. What town, city, or community do you live in? ______________________

**Basic Info: Please circle your answer.**

1. Have you heard of human papillomavirus (HPV)?  **Yes No Unsure**
2. Have you heard of cervical cancer?

**Yes No Unsure**

1. Have you or anyone you know ever been diagnosed with HPV?

**Yes No Unsure**

1. Have you or anyone you know ever been diagnosed with cervical cancer?

**Yes No Unsure**

1. Have you ever gotten a test to check for cervical cancer?

**Yes No Unsure**

6. Have you ever received the HPV vaccine?

**Yes No Unsure**

7. If you have gotten tested, **when** was the last time you were tested? ___________

**Barriers to Cervical Cancer Screening**

1. Please check off which statements apply to you.

- I am able to access cervical cancer screening easily

**If not**, please check off which reasons prevent you from accessing screening. You can check off more than one.

- I **didn’t know** cervical cancer testing is something I should get done
- It is hard for me to get **transportation** to where I need to go for the test
- The test is **too expensive** or I don’t want to spend the money on it
- There **aren’t** any **providers close by**
- I have a **religious or moral objection** to obtaining a screening
- I am **distrusting** or I feel **uncomfortable around medical providers**
- I’m **afraid** the test will **cause me pain**
- **Other reason. Please specify: ____________________________________________**

2. If a healthcare provider came to your community and offered a cervical cancer screening test at no cost would you get screened? Please circle. **Yes No Maybe**

**Cervical Cancer Knowledge Questions**: Please circle if you think the statement is true or false.

**HPV Knowledge**

- Human Papillomavirus HPV can cause cervical cancer
  - **True False Unsure**
- HPV can be passed on during sexual intercourse
  - **True False Unsure**
- Using condoms reduces the risk of getting HPV
  - **True False Unsure**
- Having many sexual partners increases the risk of getting HPV
  - **True False Unsure**
- There is a vaccine that can protect you from getting HPV
  - **True False Unsure**
- HPV vaccines are effective in preventing cervical cancer
  - **True False Unsure**

**Screening, Cervical Cancer, and Health Beliefs**

- Pap smears and acetic acid testing checks for cervical cancer
  - **True False Unsure**
- Pap smear testing checks for sexually transmitted infections
  - **True False** **Unsure**
- Cervical cancer can be prevented by Pap Smear or acetic acid testing
  - **True False Unsure**
- Cervical cancer can be cured if detected early
  - **True False Unsure**
- I believe spirits or the supernatural can influence someone’s health
  - **True False Unsure**

**Open Ended Questions:**

1. Do you have any ideas on how to increase education or cervical cancer screening in your community?

______________________________________________________________________________________________________________________________________________________________________________________________________________________________________________________________________________________________________________________________________________________________

1. Is there anything else you want to know about cervical cancer or Human Papilloma Virus (HPV)?

______________________________________________________________________________________________________________________________________________________________________________________________________________________________________________________________________________________________________________________________________________________________

**Demografía**

1. ¿Cuantos años tiene? ________ **años**
2. Por favor marque el nivel más alto de educación que haya completado.
   - Escuela primaria incompleta
   - Escuela primaria completa
   - Escuela secundaria completa
   - Escuela vocacional
   - Un poco de universidad
   - Licenciatura, maestría, doctorado, u otro título de posgrado
3. ¿En qué pueblo, ciudad, o comunidad vive? ______________________

**Información básica: Por favor dibuje un circulo en su respuesta.**

1. ¿Ha oído del virus del papiloma humano (VPH)?  **Sí No No Sabe**
2. ¿Ha oído del cáncer cervical?

**Sí No No Sabe**

1. ¿Alguna vez a usted o alguien que conoce se le ha diagnosticado con virus del papiloma humano?

**Sí No No Sabe**

1. ¿Alguna vez a usted o alguien que conoce se le ha diagnosticado con cáncer cervical?

**Sí No No Sabe**

1. ¿Alguna vez se le ha realizado una prueba para detectar el cáncer cervical?

**Sí No No Sabe**

6. ¿Alguna vez ha recibido la vacuna contra el virus del papiloma humano?

**Sí No No Sabe**

7. Si le hicieron la prueba, ¿**cuándo** fue la última vez que se la hicieron? ___________

**Barreras contra la Detección del Cáncer Cervical**

1. Por favor marque qué declaraciones le aplican a usted.

- Puedo acceder a la prueba del cáncer cervical fácilmente

**Si no**, por favor marque las razones que le impiden acceder a la prueba de detección. Puedes marcar más de uno.

- **No sabía** que las pruebas del cáncer cervical eran algo que debería hacer
- Es difícil para mí conseguir **transporte** hacia el lugar donde necesito ir para la prueba
- La prueba es **demasiado costosa** o no quiero gastar el dinero en dicha prueba
- **No hay** ningún **proveedor cerca**
- Tengo una **objeción religiosa o moral** para obtener una prueba
- **Desconfío** o me siento **incómoda con los proveedores médicos**
- Tengo **miedo** de que la prueba **me cause dolor**.
- **Otra razón. Por favor especifica: ____________________________________________**

2. Si un proveedor de atención médica viniera a su comunidad y le ofreciera una prueba de detección de cáncer cervical sin ningún costo, ¿se haría la prueba? Por favor marque.

**Si No Tal Vez**

**Preguntas sobre el Conocimiento del Cáncer Cervical:** por favor dibuje un circulo indicando si piensa que la declaración es verdadera o falsa.

**Conocimiento del virus del papiloma humano**

1. El virus del papiloma humano virus del papiloma humano puede causar cáncer cervical

**Cierto Falso No Sabe**

2. El virus del papiloma humano se puede transmitir durante las relaciones sexuales

**Cierto Falso No Sabe**

3. Usando condones reduce el riesgo de contraer el virus del papiloma humano

**Cierto Falso No Sabe**

4. Tener muchas parejas sexuales aumenta el riesgo de contraer el virus del papiloma humano

**Cierto Falso No Sabe**

5. Hay una vacuna que puede protegerla de contraer el virus del papiloma humano

**Cierto Falso No Sabe**

6. Las vacunas contra el virus del papiloma humano son efectivas en la prevención del cáncer cervical

**Cierto Falso No Sabe**

**Pruebas, Cáncer Cervical, y Creencias Sobre la Salud**

7. Las pruebas de Papanicolaou y las pruebas de ácido acético detectan el cáncer cervical

**Cierto Falso No Sabe**

8. Las pruebas de Papanicolaou detectan infecciones de transmisión sexual

**Cierto Falso No Sabe**

9. El cáncer cervical se puede prevenir mediante la prueba de Papanicolaou o la prueba de ácido acético

**Cierto Falso No Sabe**

10. El cáncer cervical se puede curar si se detecta a tiempo

**Cierto Falso No Sabe**

11. Creo que los espíritus o lo sobrenatural puede influir en la salud de alguien

**Cierto Falso No Sabe**

**Preguntas Abiertas:**

1. ¿Tiene alguna idea de cómo aumentar la educación o las pruebas del cáncer cervical en su comunidad?

______________________________________________________________________________________________________________________________________________________________________________________________________________________________________________________________________________________________________________________________________________________________

2. ¿Hay algo más que desea saber sobre el cáncer cervical o el Virus del Papiloma Humano (VPH)?

______________________________________________________________________________________________________________________________________________________________________________________________________________________________________________________________________________________________________________________________________________________________
