# Supplementary material for: Knowledge of Cervical Cancer Prevention Among Women in Amazonian Peru
Source: Womens Health Rep (New Rochelle). 2020 Aug 17;1(1):270–8. doi: 10.1089/whr.2020.0051 (PMC7784820; doi:10.1089/whr.2020.0051)
Supplement: Supplemental data [file Supp_Data.zip › 0051 Gochenaur&Peterson.AppendixII.docx]

CERVICAL CANCER


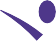

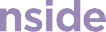


About Gynecologic Cancer

There are five main types of cancer that affect a woman’s reproductive organs: cervical, ovarian, uterine, vaginal, and vulvar. As a group, they are referred to as gynecologic (GY-neh-kuh-LAH-jik) cancer. (A sixth type of gynecologic cancer is the very rare fallopian tube cancer.)

This fact sheet about cervical cancer is part of the Centers for Disease Control and Prevention’s (CDC) *Inside Knowledge: About Gynecologic Cancer* campaign. The campaign helps women get the facts about gynecologic cancer, providing important “inside knowledge” about their bodies and health.

## What is cervical cancer?

Cancer is a disease in which cells in the body grow out of control.

Cancer is always named for the part of the body where it starts, even if it spreads to other body parts later.

When cancer starts in the

cervix, it is called cervical cancer. The cervix is the lower, narrow end of the uterus. The cervix

connects the vagina (the birth canal) to the upper part of the uterus. The uterus (or womb) is where a baby grows when a woman is pregnant.

Cervical cancer is the easiest gynecologic cancer to prevent

## Are there tests that can prevent cervical cancer or find it early?

There are two tests that can either help prevent cervical cancer or find it early:

- Depending on your age, your doctor may recommend you have a Pap test, or an HPV test, or both tests together.
- The Pap test (or Pap smear) looks for precancers, cell changes, on the cervix that can be treated, so that cervical cancer is prevented. The Pap test also can find cervical cancer early, when treatment is most effective.

The Pap test only screens for cervical cancer. It does not screen for any other gynecologic cancer.

- The HPV test looks for HPV—the virus that can cause precancerous cell changes and cervical cancer.

with regular screening tests and follow-up. It also is highly curable when found and treated early.

## Who gets cervical cancer?

All women are at risk for cervical cancer. It occurs most often in women over age 30. Each year, approximately 12,000 women in the United States get cervical cancer.


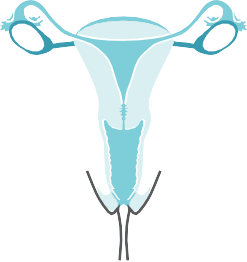


The human papillomavirus (HPV) is the main cause of cervical

Fallopian Tube Ovary

Uterus **Cervix** Vagina Vulva

cancer. HPV is a common virus

that is passed from one person to another during sex. Most sexually active people will have HPV at some point in their lives, but few women will get cervical cancer.

## What are the symptoms?

Early on, cervical cancer may not cause signs and symptoms.

Advanced cervical cancer may cause bleeding or discharge from the vagina that is not normal for you, such as bleeding after sex. If you have any of these signs, see your doctor. They may be caused by something other than cancer, but the only way to know is to see your doctor.

# [cdc.gov/cancer/knowledge](https://www.cdc.gov/cancer/knowledge) 800-CDC-INFO


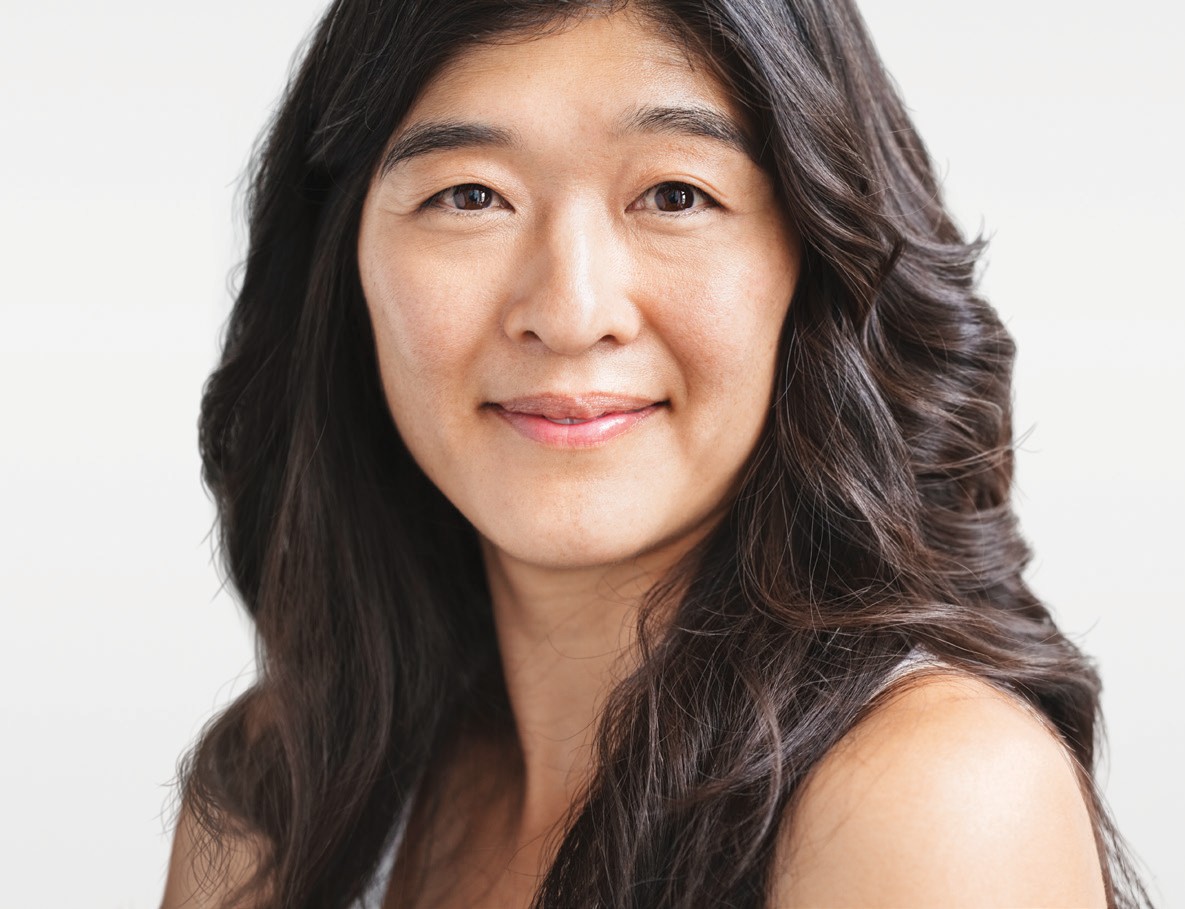


## When should I get tested for cervical cancer?

The Pap test is one of the most reliable and effective cancer screening tests available. The Pap test is recommended for all women between the ages of 21 and 29 years old. If your Pap test results are normal, your doctor may say that you will not need another Pap test for three years.

If you are 30 years old or older, you may choose to have a Pap test, or an HPV test, or both tests together. If the results are normal, your chance of getting cervical cancer in the next few years is very low. Your doctor may then say that you can wait up to five years for your next screening.

The HPV test is also used to provide more information when women aged 21 years or older have unclear Pap test results.

For women aged 21-65, it is important to continue getting a Pap and/or HPV test as directed by your doctor—even if you think you are too old to have a child

or are not having sex anymore. However, your doctor may tell you that you do not need to have a Pap or HPV test if either of these is true for you:

- You are older than 65 and have had a normal Pap or HPV test for several years.
- You have had your cervix removed as part of a total hysterectomy for

non-cancerous conditions, like fibroids.

## What raises a woman’s chance of getting cervical cancer?

Almost all cervical cancers are caused by HPV. You are more likely to get HPV if you started having sex at an early age, or if you or your partner have had sex with several others. However, any woman who has ever had sex is at risk for HPV.

There are many types of HPV. Usually HPV will go away on its own, but if it does not, it may cause cervical cancer over time.

In addition to having HPV, these things also can increase your risk of cervical cancer:

- Smoking.
- Having HIV (the virus that causes AIDS) or another condition that makes it hard for your body to fight off health problems.
- Using birth control pills for a long time (five or more years).
- Having given birth to three or more children.

## How can I prevent cervical cancer?

- See your doctor regularly for a Pap and/or HPV test.
- Follow up with your doctor if your cervical cancer screening test results are not normal.
- Get the HPV vaccine. It protects against the types of HPV that most often cause cervical, vaginal, and vulvar cancers. It

is recommended for preteens (both boys and girls) aged 11 to 12 years, but can be given as early as age 9 and until age

26. The vaccine is given in a series of either two or three shots, depending on age. It is important to note that even

women who are vaccinated against HPV need to have regular Pap tests to screen for cervical cancer. To learn more about the HPV vaccine visit [**www.cdc.gov/hpv**.](https://www.cdc.gov/hpv/)

- Don’t smoke.
- Use condoms during sex.*
- Limit your number of sexual partners.
- HPV infection can occur in both male and female genital areas that are covered or

protected by a latex condom, as well as in areas that are not covered. While the effect of condoms in preventing HPV infection is unknown, condom use has been associated with a lower rate of cervical cancer.

## What should I do if my doctor says I have cervical cancer?

If your doctor says that you have cervical cancer, ask to be referred to a gynecologic oncologist—a doctor who has been trained to treat cancers like this. This doctor will work with you to create a treatment plan.

## Where can I find free or low-cost cervical cancer screening tests?

If you have a low income or do not have insurance, you may be able to get a free or low-cost cervical cancer screening test through the

National Breast and Cervical Cancer Early Detection Program. To learn more, call 800-CDC-INFO or visit [www.cdc.gov/cancer/nbccedp.](https://www.cdc.gov/cancer/nbccedp/)

## Where can I find more information about cervical and other gynecologic cancers?

Centers for Disease Control and Prevention: 800-CDC-INFO or [www.cdc.gov/cancer/gynecologic](https://www.cdc.gov/cancer/gynecologic/)

National Cancer Institute:

800-4-CANCER or [www.cancer.gov](https://www.cancer.gov/)

#
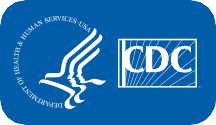

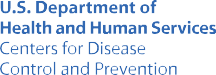
[cdc.gov/cancer/knowledge](https://cdc.gov/cancer/knowledge) 800-CDC-INFO


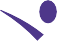

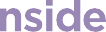


About Gynecologic Cancer

*CDC Publication #99-9123, Revised January 2019*


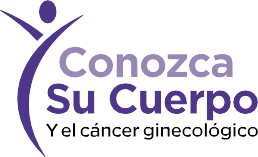
CÁNCER DE CUELLO UTERINO

Existen cinco tipos principales de cáncer que afectan los órganos reproductores de la mujer: los cánceres de cuello uterino, ovario, útero, vagina y vulva.

En conjunto se les conoce como cáncer ginecológico. (Un sexto tipo de cáncer ginecológico poco frecuente es el cáncer de las trompas de Falopio).

Esta hoja informativa sobre el cáncer de cuello uterino es parte de la campaña *Conozca su cuerpo: Y el cáncer ginecológico* de los Centros para el Control y la Prevención de Enfermedades (CDC por sus siglas en inglés). La campaña ayuda a que las mujeres aprendan sobre el cáncer ginecológico y brinda importante información acerca de sus cuerpos y salud.

### **¿Qué es el cáncer de cuello uterino?**

El cáncer es una enfermedad que provoca el crecimiento descontrolado de las células. El tipo de cáncer siempre se identifica según la parte del cuerpo en donde se origina, incluso si después se extiende a otras partes del cuerpo.

Cuando el cáncer se origina en el cuello uterino, se denomina como cáncer de cuello uterino. El cuello uterino es la parte más baja y estrecha del útero. El cuello uterino conecta la parte superior del útero con la vagina (vía del parto). El útero (o matriz) es

el lugar donde se desarrolla el bebé cuando una mujer está embarazada.

El cáncer de cuello uterino es el cáncer ginecológico más fácil de prevenir con pruebas regulares de detección y seguimiento. Además, es altamente curable cuando se detecta y se trata en etapas tempranas.

**¿Quién puede contraer cáncer de cuello uterino?**

Todas las mujeres corren riesgo de contraer cáncer de cuello uterino. Ésta enfermedad afecta con mayor frecuencia a mujeres mayores de 30 años y en Estados Unidos, se estima que 12,000 mujeres lo contraen cada año. La causa principal es el virus del papiloma humano (VPH). El VPH es un virus común que puede transmitirse de una persona a otra durante las relaciones sexuales. Aunque la mayoría de las personas sexualmente activas estarán infectadas por el VPH en algún momento de su vida, pocas mujeres contraerán cáncer de cuello uterino.

### **¿Cuáles son los síntomas?**

En etapas tempranas, el cáncer de cuello uterino no suele presentar signos ni síntomas. Cuando ya está avanzado puede producir sangrado o secreción vaginal anormal, como por ejemplo, sangrado después


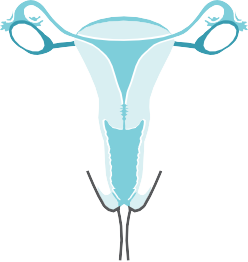


de tener relaciones sexuales.

Trompa de Falopio Ovario

Útero

**Cuello uterino**

Vagina Vulva

### **¿Existen pruebas que podrían prevenir o detectar el cáncer de cuello uterino en sus etapas tempranas?**

Existen dos pruebas que pueden ayudar a prevenirlo o detectarlo en etapas tempranas:

- Dependiendo de su edad, su médico podría recomendarle que se haga la prueba de Papanicolaou, VPH o ambas pruebas juntas.
- La prueba de Papanicolaou (o citología vaginal) busca

precánceres, cambios celulares en el cuello uterino que pueden ser tratados para prevenir

este tipo de cáncer. La prueba de Papanicolaou también puede detectar el cáncer de cuello uterino en etapas tempranas cuando el tratamiento es

más eficaz.

La prueba de Papanicolaou solo detecta el cáncer de cuello uterino. No detecta ningún otro tipo de cáncer ginecológico.

- La prueba de VPH busca el virus del papiloma humano, el virus que puede causar cambios celulares y cáncer de cuello uterino.

# cdc.gov/spanish/cancer/knowledge 800-CDC-INFO


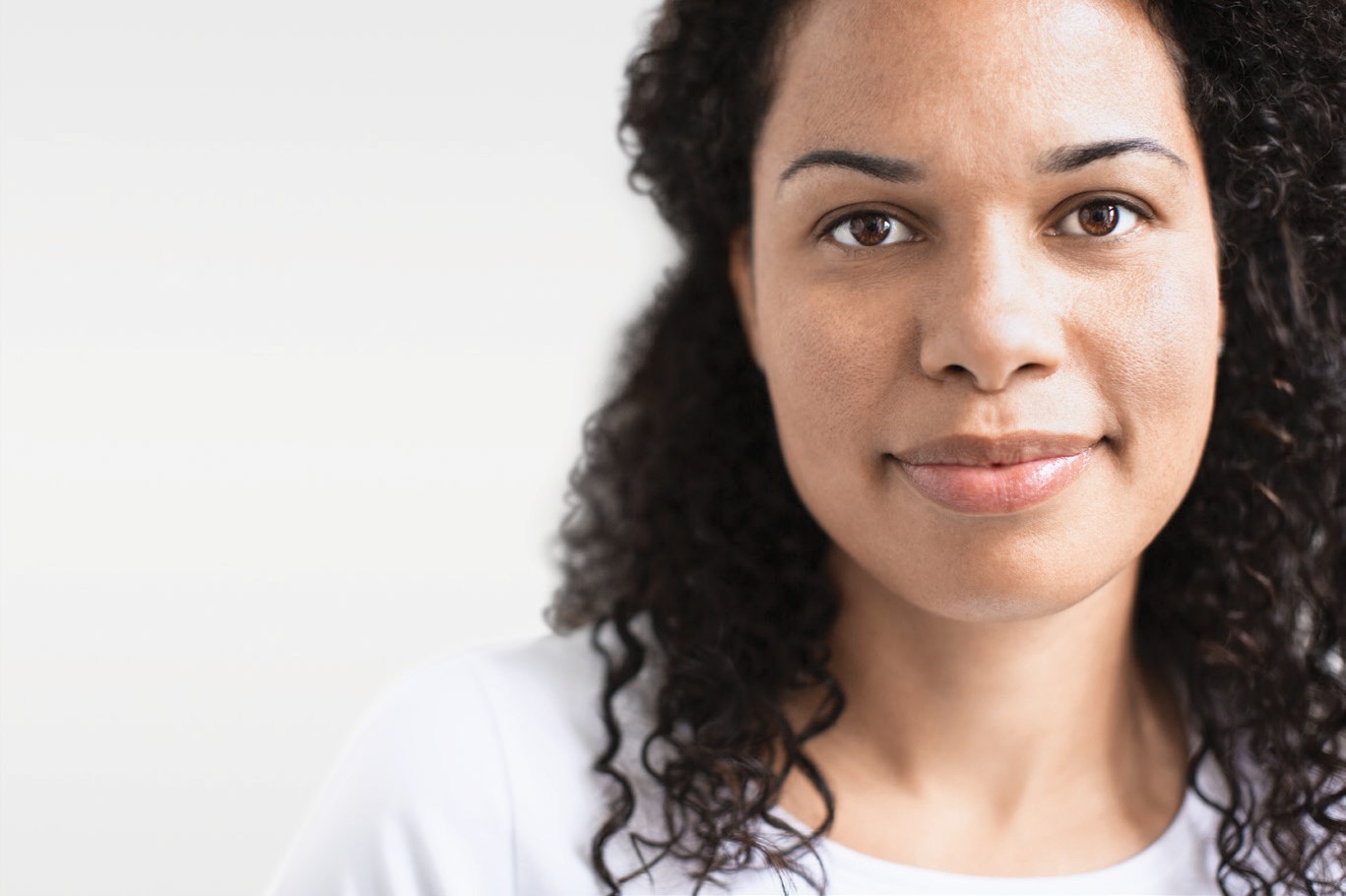


### **¿Cuándo debo hacerme la prueba de detección del cáncer de cuello uterino?**

La prueba de Papanicolaou es una de las pruebas de detección más confiables y efectivas disponibles. Ésta prueba es recomendada para todas las mujeres entre 21 y 29 años. Si sus resultados son normales, es posible que su médico le diga que no necesita hacerse otra prueba de Papanicolaou durante los próximos tres años.

Si tiene 30 años o más, usted puede elegir hacerse una prueba de Papanicolaou, de VPH o ambas pruebas juntas. Si los resultados son normales, su probabilidad de contraer cáncer de cuello uterino en los próximos años es mínima. Si es así, su médico puede decirle que

puede esperar hasta cinco años para su próxima prueba de detección. La prueba de VPH también se usa para proporcionar información adicional cuando las mujeres de 21 años o más obtienen resultados de Papanicolaou poco claros.

Es importante que las mujeres de 21-65 años continúen haciéndose la

prueba de Papanicolaou y/o de VPH según las indicaciones del médico, aún cuando piensen que ya no están en edad para tener hijos o no sean sexualmente activas.

Sin embargo, su médico podría decirle que ya no necesita hacerse las pruebas de Papanicolaou o de VPH

si alguno de estos factores es cierto para usted:

- Es mayor de 65 años y sus resultados de la prueba de Papanicolaou o VPH han sido normales durante varios años.
- Le han extirpado el cuello uterino como parte de una histerectomía total debido a problemas no cancerosos, como fibromas.

Si tiene cualquiera de estos síntomas, consulte a su médico. Podrían ser ocasionados por alguna otra causa, pero pero es mejor averiguar.

### **¿Qué aumenta la probabilidad de que una mujer contraiga cáncer de cuello uterino?**

El cáncer de cuello uterino casi siempre es causado por el VPH. Usted tiene mayor probabilidad de contraer el VPH si comenzó a tener relaciones sexuales a una edad temprana o si usted o su pareja han tenido relaciones sexuales con varias personas. Sin embargo, toda mujer que ha tenido relaciones sexuales alguna vez corre el riesgo de contraer el VPH. Existen muchos tipos de VPH. Por lo general, el VPH desaparece por sí solo pero si permanece puede causar cáncer de cuello uterino. Además del VPH, los siguientes factores pueden aumentar el riesgo de contraer cáncer de cuello uterino:

- Fumar.
- Tener el VIH (el virus que causa el SIDA) u otra condición médica que haga difícil que su cuerpo combata problemas de salud.
- Usar píldoras anticonceptivas por un largo tiempo (cinco años o más).
- Haber dado a luz a tres o más niños.

### **¿Cómo puedo prevenir el cáncer de cuello uterino?**

- Visite a su médico con regularidad para una prueba de Papanicolaou y/o de VPH.
- Si los resultados son anormales, hable con su médico.
- Póngase la vacuna contra el VPH. Esta vacuna protege contra los tipos de VPH que causan el cáncer de cuello uterino, vagina y vulva. Se recomienda para preadolescentes (niños y niñas) de 11 a 12 años pero puede aplicarse desde los 9 9 y hasta los 26 años. La vacuna es administrada en dosis de dos o tres inyecciones, dependiendo

de la edad. Es importante que las mujeres se hagan la prueba de Papanicolaou frecuentemente

para detectar el cáncer de cuello uterino aunque se hayan puesto

la vacuna contra el VPH. Para más información sobre esta vacuna visite: [**www.cdc.gov/spanish/cancer/**](http://www.cdc.gov/spanish/cancer/) **hpv/index.htm.**

- No fume.
- Use condones durante las relaciones sexuales.*
- Limite el número de sus parejas sexuales.
- La infección por el VPH puede afectar los órganos genitales tanto de varones como de mujeres que están

protegidos o cubiertos por un condón de látex, así como las áreas descubiertas. Aunque se desconoce el efecto de los condones para prevenir la infección

por el VPH, su uso se ha relacionado con una tasa más baja de cáncer de cuello uterino.

### **¿Qué debo hacer si el médico me dice que tengo cáncer de cuello uterino?**

Si su médico le dice que tiene cáncer de cuello uterino, pídale que la remita a un ginecólogo oncólogo, un médico especializado en el tratamiento de cáncer ginecológico. Este médico trabajará con usted para crear un plan de tratamiento.

### **¿Dónde puedo encontrar pruebas de detección para el cáncer de cuello uterino gratuitas o de bajo costo?**

Si tiene bajos ingresos o no tiene seguro médico, podría obtener una prueba de detección de cáncer de cuello uterino gratuita o de bajo costa a través del Programa Nacional de Detección Temprana del Cáncer de Mama y de Cuello Uterino.

Para más información, llame al

1-800-CDC-INFO o visite www.cdc. gov/spanish/cancer/dcpc/about/ nbccedp.htm.

### **¿Dónde puedo encontrar más información sobre el cáncer de cuello uterino y otros cánceres ginecológicos?**

Centros para el Control y la Prevención de Enfermedades:

1-800-CDC-INFO (1-800-232-4636)

Oprima 2 para español, o visite [www.](http://www/) cdc.gov/spanish/cancer/gynecologic.

Instituto Nacional del Cáncer:

1-800-4-CANCER (1-800-422-6237)

Oprima 2 para español, o visite [www.cancer.gov/espanol.](http://www.cancer.gov/espanol)


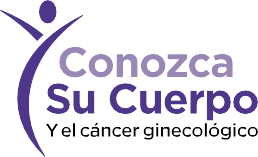
cdc.gov/spanish/cancer/knowledge 800-CDC-INFO


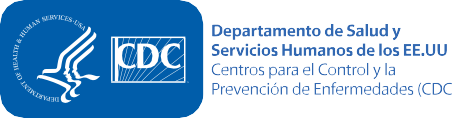


*Publicación de los CDC No 99-9123, revisada en enero de 2019*
